# Supplementary material for: CO2 subsurface mineral storage by its co-injection with recirculating water
Source: Nature. 2026 Mar 25;651(8107):954–8. doi: 10.1038/s41586-026-10130-5 (PMC13017520; doi:10.1038/s41586-026-10130-5)
Supplement: Supplementary file 1 — This file contains Supplementary Fig. 1 containing diffraction patterns of samples described in Extended Data Table 3, Supplementary Tables 1–3 providing the measured fluid sample compositions and mineral saturation states and Supplementary Table 4 listing the powder diffraction files to analyse XRD images [file 41586_2026_10130_MOESM1_ESM.pdf]

nature portfolio

https://doi.org/10.1038/s41586-026-10130-5

Supplementary information

CO_2 subsurface mineral storage by its co-injection with recirculating water

In the format provided by the authors and unedited

Supplementary Information

a) Sample 9-A

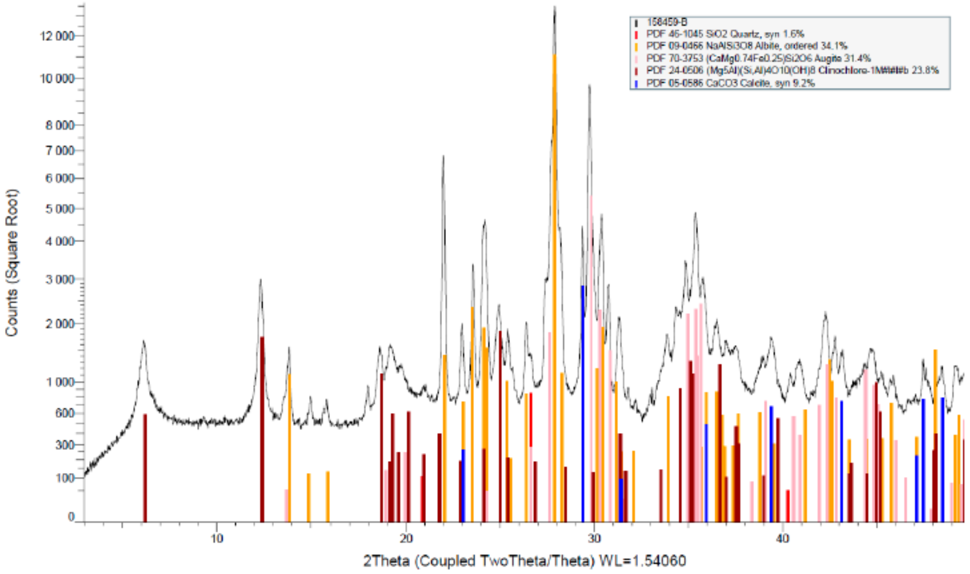

b) Sample 9-B

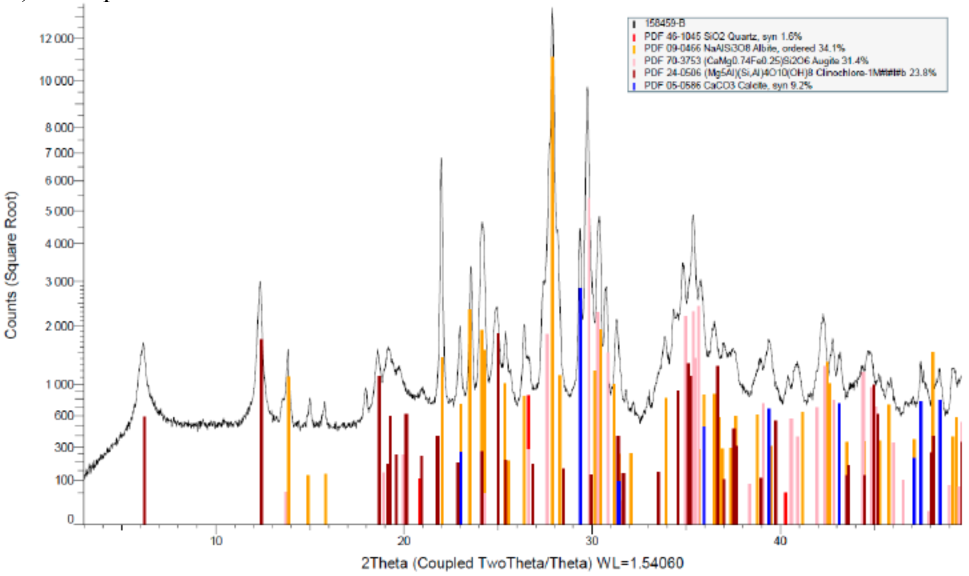

Supplementary Figure 1. | X-ray diffraction patterns obtained of the solids collected from the collected from inside the damaged submersible pump on September 10, 2023. Mineral compositions obtained from these patterns are provided in weight percent in Extended Data Table 3.

c) Sample 9D

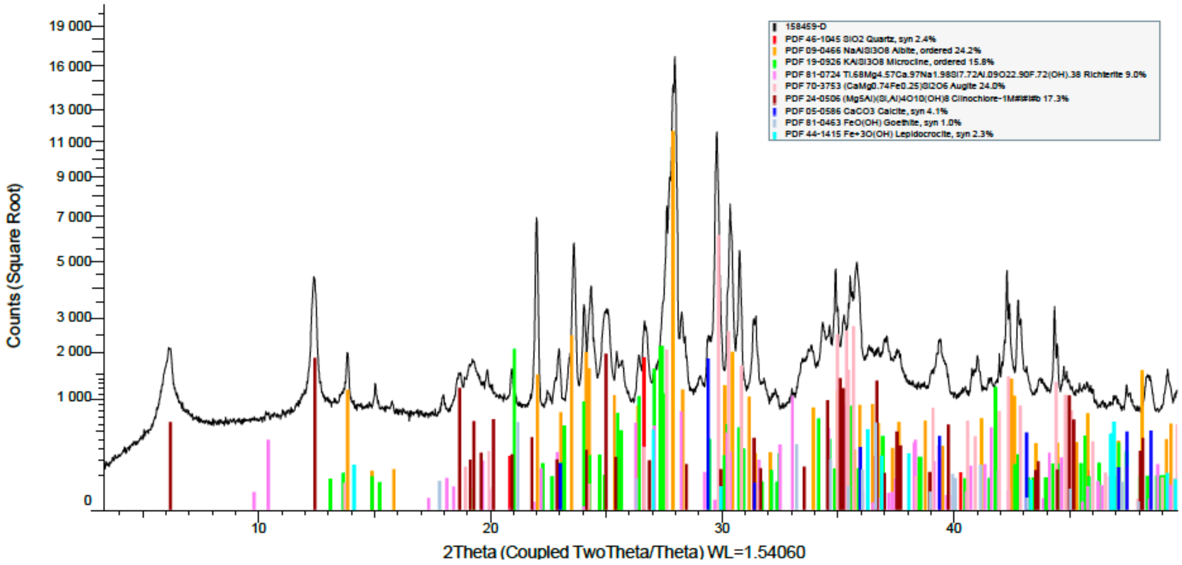

d) Sample 9-E

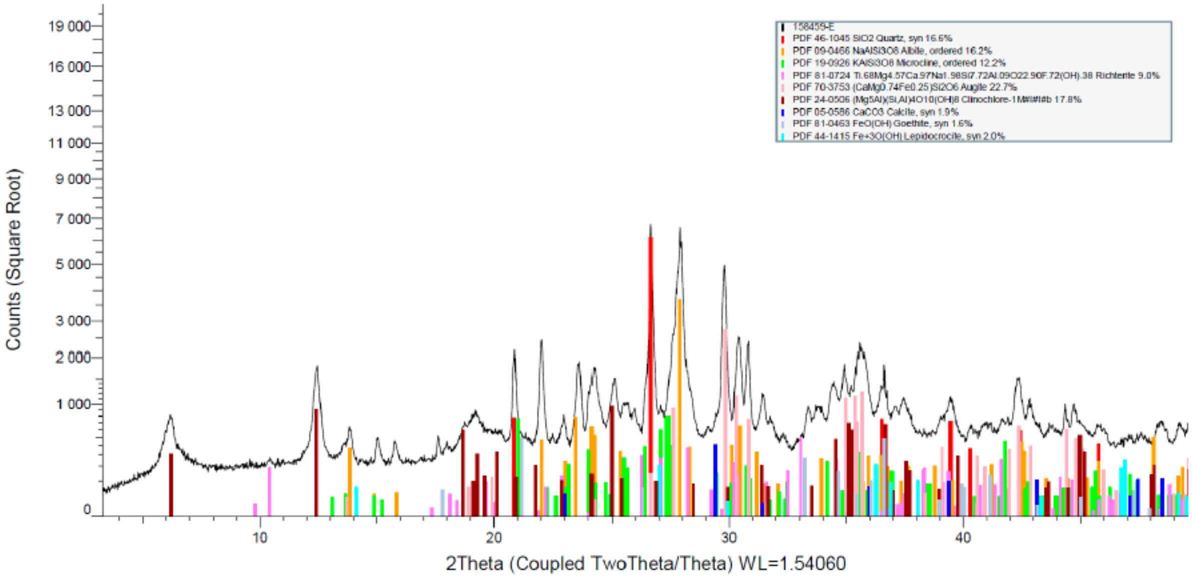

Supplementary Figure 1. (continued). | X-ray diffraction patterns obtained of the solids collected from the collected from inside the damaged submersible pump on September 10, 2023. Mineral compositions obtained from these patterns are provided in weight percent in Extended Data Table 3.

e) Sample 9-F

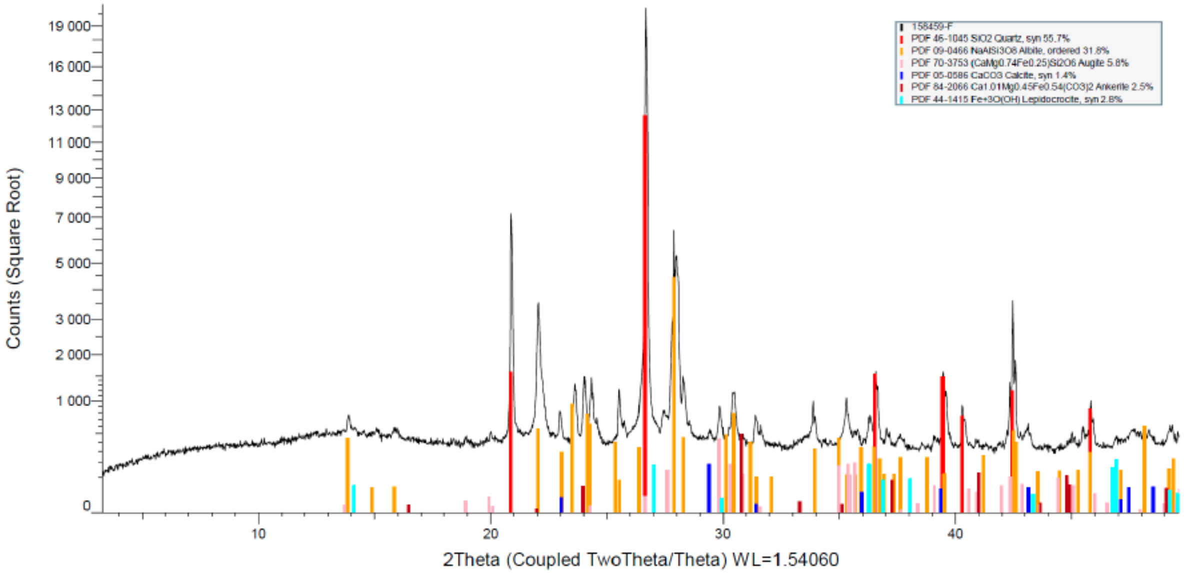

f) Sample 9-G

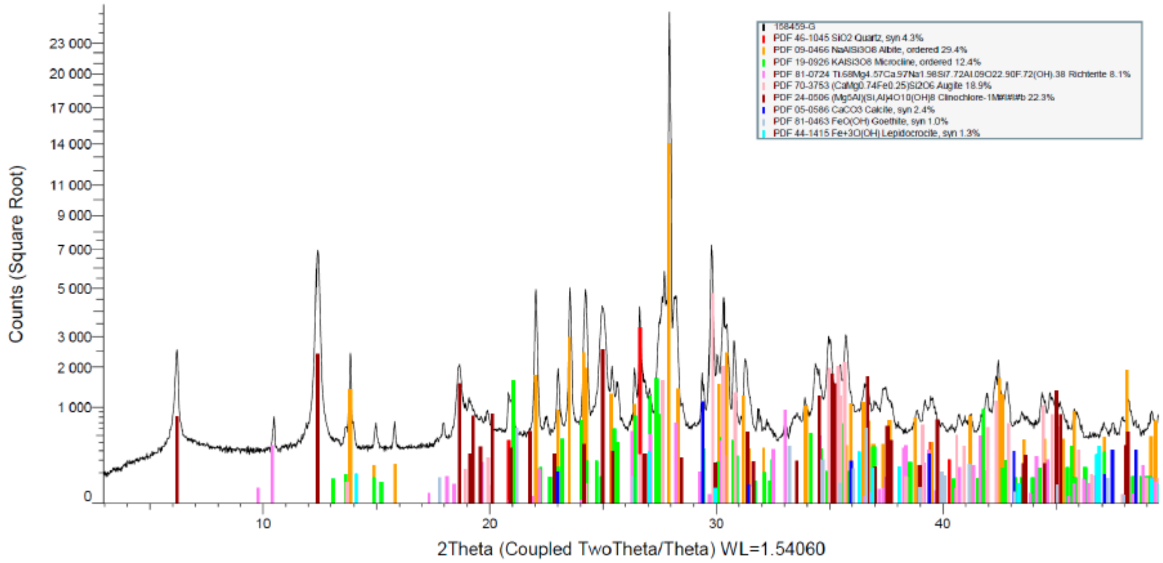

Supplementary Figure 1. (continued). | X-ray diffraction patterns obtained of the solids collected from the collected from inside the damaged submersible pump on September 10, 2023. Mineral compositions obtained from these patterns are provided in weight percent in Extended Data Table 3.

g) Sample 9-H

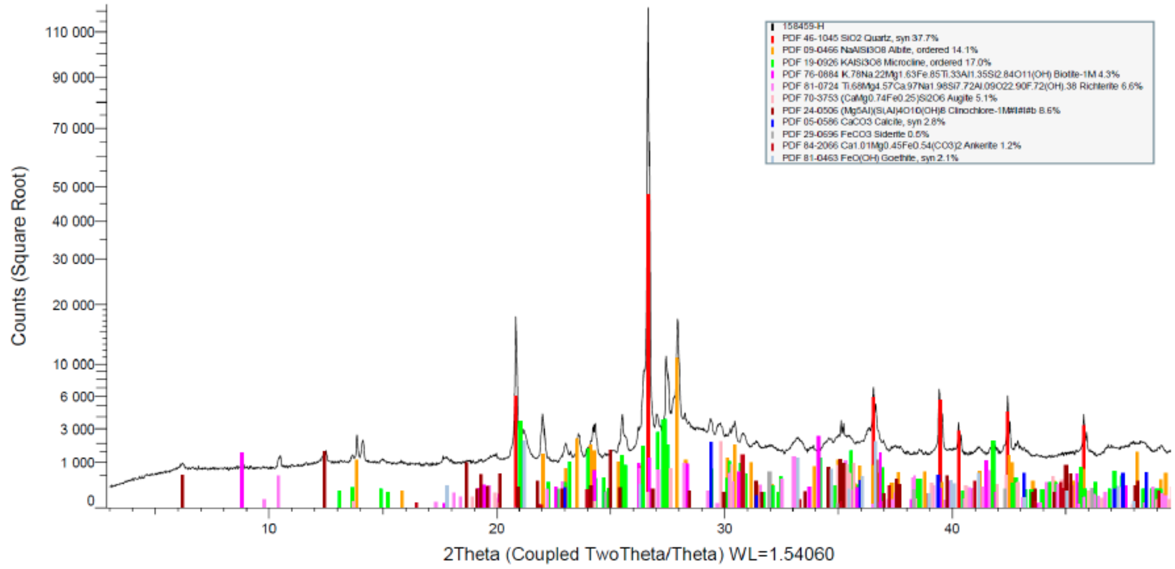

h) Sample 9-J

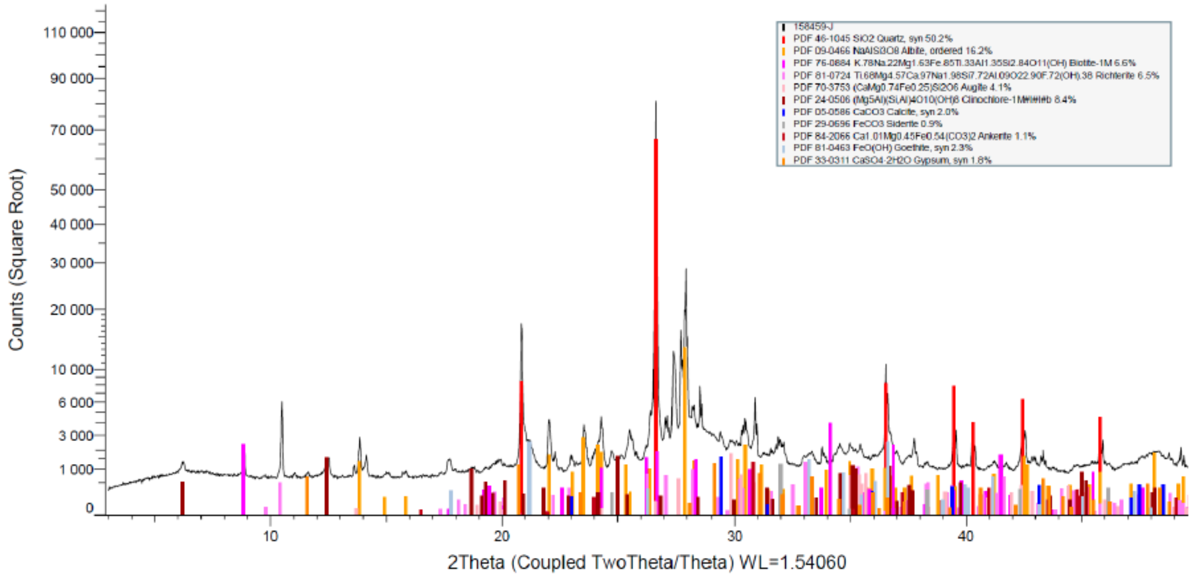

Supplementary Figure 1. (continued). | X-ray diffraction patterns obtained of the solids collected from the collected from inside the damaged submersible pump on September 10, 2023. Mineral compositions obtained from these patterns are provided in weight percent in Extended Data Table 3.

i) Sample 9-K

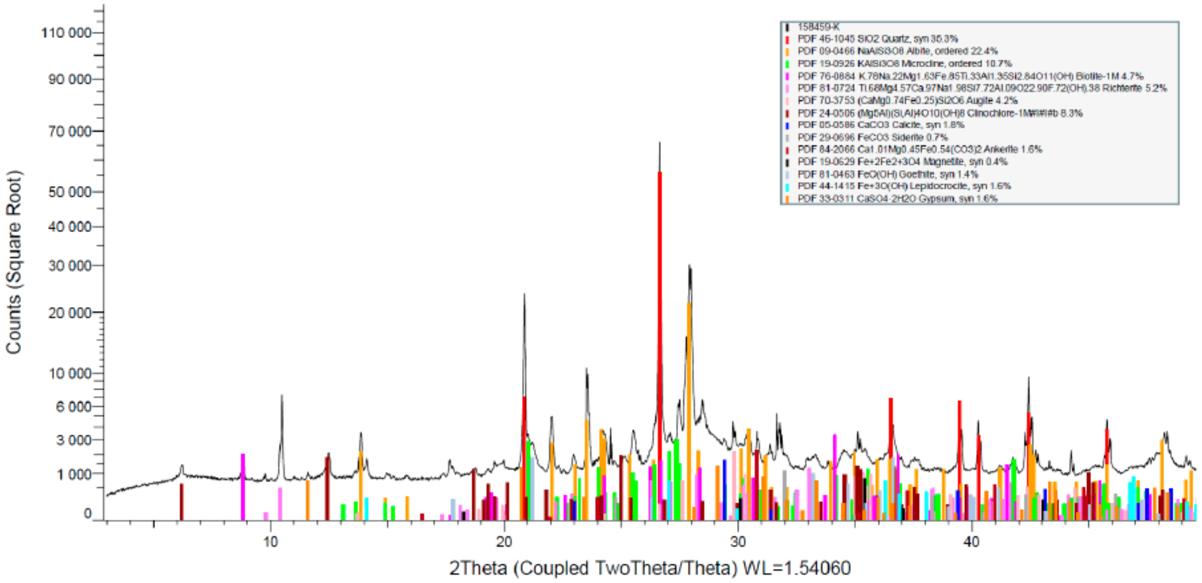

j) Sample 9-L

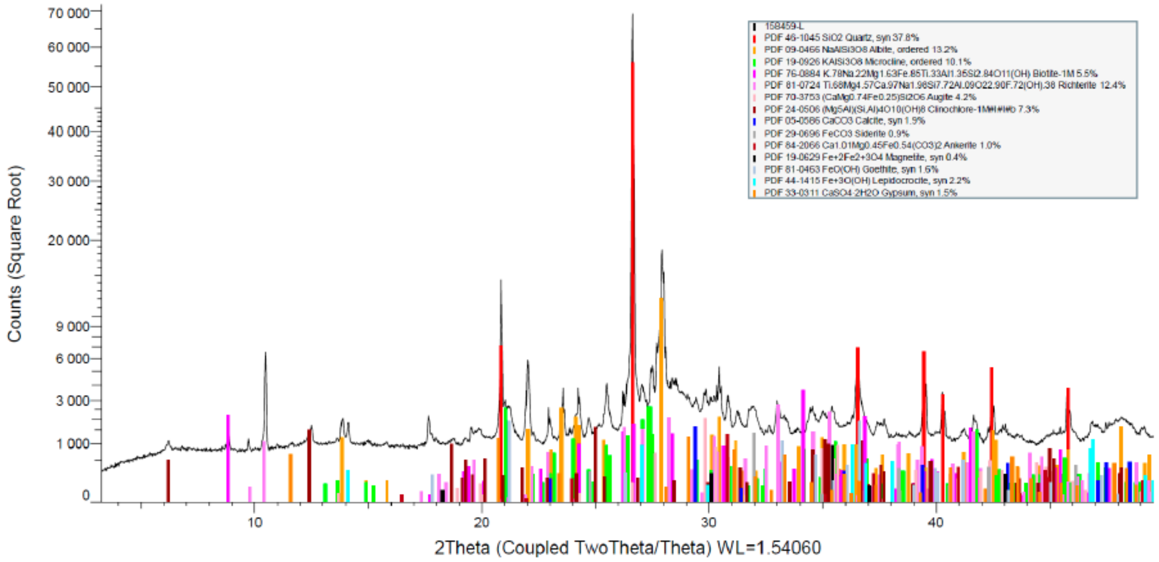

Supplementary Figure 1. (continued). | X-ray diffraction patterns obtained of the solids collected from the collected from inside the damaged submersible pump on September 10, 2023. Mineral compositions obtained from these patterns are provided in weight percent in Extended Data Table 3.

k) Sample 9-M

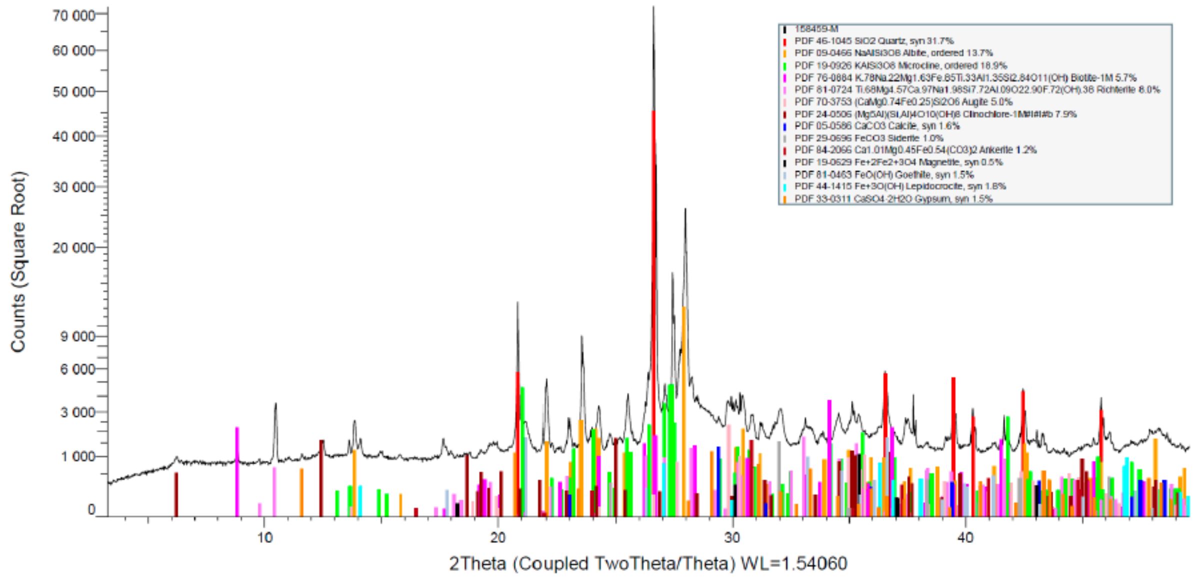

l) Sample 9-N

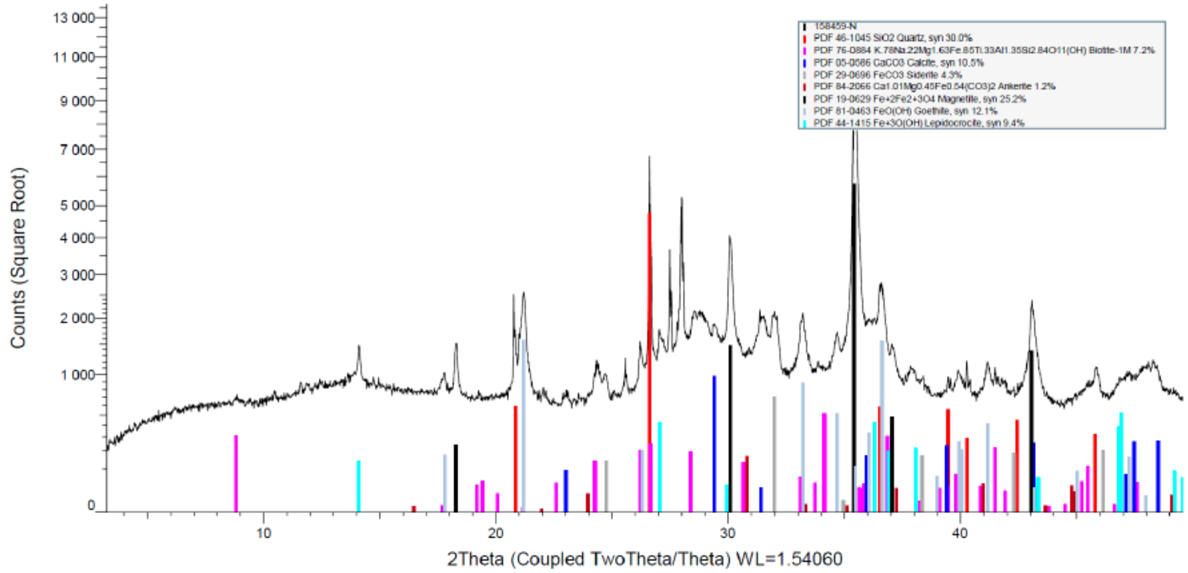

Supplementary Figure 1. (continued). | X-ray diffraction patterns obtained of the solids collected from the collected from inside the damaged submersible pump on September 10, 2023. Mineral compositions obtained from these patterns are provided in weight percent in Extended Data Table 3.

m) Sample 9-O

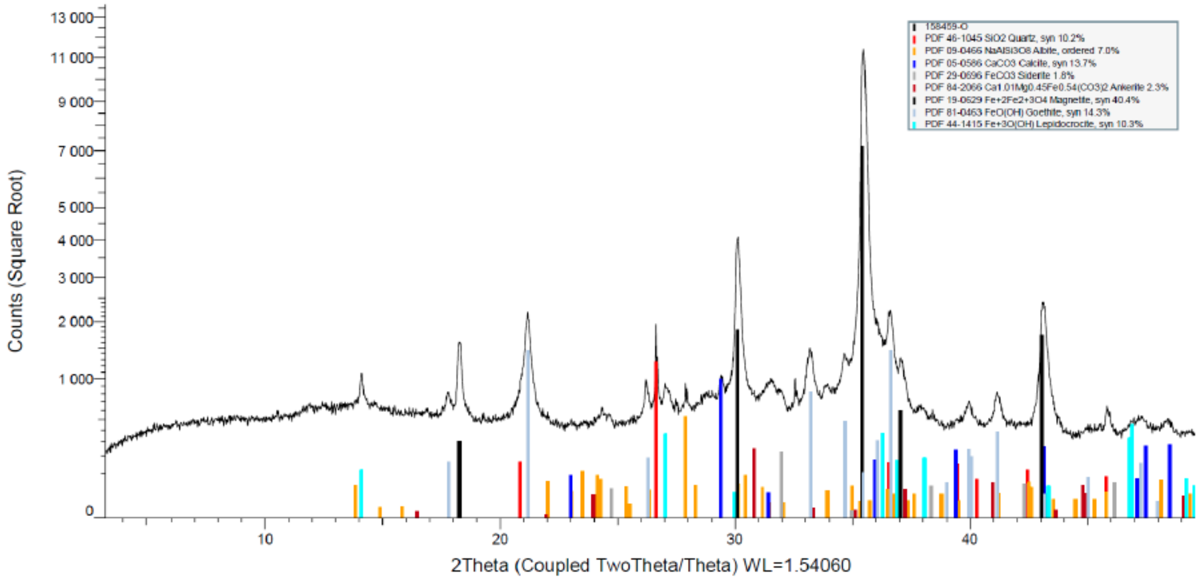

Supplementary Figure 1. (continued). | X-ray diffraction patterns obtained of the solids collected from the collected from inside the damaged submersible pump on September 10, 2023. Mineral compositions obtained from these patterns are provided in weight percent in Extended Data Table 3.

Supplementary Table 1 | Measured composition of collected fluid samples from May 20, 2023 until April 21, 2024. The concentrations of the elements are provided in units of mmol kg^{-1}, whereas the alkalinity is reported in units of meq kg^{-1}. BD indicates the concentration was below the detection limit of the analysis.

Date	Ca	Fe	K	Mg	Na	Si	Alkalinity
20-May	19.02	BD	0.27	0.42	27.08	0.55	0.32
23-May	19.30	BD	0.28	0.44	27.31	0.56	
24-May	19.46	BD	0.27	0.42	27.53	0.56	
26-May	19.94	BD	0.29	0.44	28.43	0.63	
29-May	19.50	BD	0.27	0.43	27.57	0.59	
1-Jun	19.90	BD	0.28	0.44	28.03	0.61	0.65
3-Jun	19.26	BD	0.27	0.44	27.40	0.62	
5-Jun	19.74	BD	0.28	0.45	27.79	0.65	0.80
10-Jun	19.68	BD	0.27	0.47	27.73	0.66	0.84
11-Jun	19.92	BD	0.26	0.44	27.77	0.66	
13-Jun	19.77	BD	0.28	0.47	27.72	0.70	1.17
15-Jun	20.23	BD	0.28	0.49	28.05	0.71	
17-Jun	20.03	BD	0.27	0.47	27.88	0.72	1.57
19-Jun	20.10	BD	0.27	0.50	27.82	0.76	2.09
21-Jun	20.35	BD	0.28	0.51	27.94	0.79	2.54
22-Jun	20.43	BD	0.27	0.53	27.80	0.80	
24-Jun	20.91	0.002	0.27	0.54	28.08	0.84	3.53
27-Jun	20.81	0.003	0.29	0.60	27.97	0.89	4.06
29-Jun	21.22	0.007	0.29	0.60	28.16	0.93	
1-Jul	21.35	0.012	0.29	0.66	28.23	0.97	5.26
3-Jul	21.81	0.014	0.28	0.65	28.62	0.97	
5-Jul	22.03	0.016	0.29	0.70	28.50	1.02	6.18
7-Jul	22.49	0.019	0.28	0.74	29.16	1.02	6.75
10-Jul	22.54	0.023	0.29	0.77	28.72	1.06	
14-Jul	22.50	0.025	0.30	0.88	28.75	1.13	8.23
18-Jul	23.07	0.027	0.30	0.95	29.94	1.16	9.15
22-Jul	23.12	0.026	0.30	0.96	29.92	1.17	9.80
26-Jul	23.61	0.026	0.30	1.04	30.09	1.19	10.03
30-Jul	23.43	0.032	0.30	1.03	30.27	1.19	10.40
3-Aug	23.85	0.028	0.30	1.10	30.45	1.22	10.33
7-Aug	23.63	0.030	0.30	1.09	30.40	1.21	9.82
11-Aug	23.68	0.027	0.30	1.11	30.53	1.23	10.00
15-Aug	23.64	0.025	0.30	1.15	30.52	1.21	9.58
19-Aug	23.21	0.026	0.30	1.12	30.25	1.21	9.11
23-Aug	23.41	0.029	0.30	1.15	30.24	1.22	8.99
27-Aug		0.028	0.30	1.13		1.21	8.72

Supplementary Table 1 (continued) | Measured composition of collected fluid samples from May 20, 2023 until April 21, 2024. The concentrations of the elements are provided in units of mmol kg^{-1}, whereas the alkalinity is reported in units of meq kg^{-1}

Date	Ca	Fe	K	Mg	Na	Si	Alkalinity
4-Sep	23.33	0.029	0.29	1.11	30.16	1.18	8.28
8-Sep	23.01	0.028	0.29	1.13	29.81	1.20	7.86
2-Oct	23.43	0.033	0.29	1.30	30.05	1.12	8.36
6-Oct	20.56	0.031	0.27	1.16	27.94	1.09	6.96
10-Oct	20.76	0.032	0.28	1.19	27.94	1.13	6.55
14-Oct	21.14	0.034	0.29	1.15	28.70	1.15	
18-Oct	21.10	0.033	0.28	1.15	28.41	1.14	6.24
22-Oct	20.84	0.034	0.30	1.16	29.42	1.17	6.17
26-Oct	20.86	0.034	0.30	1.19	29.47	1.18	
30-Oct	20.39	0.033	0.30	1.16	28.97	1.17	5.96
3-Nov	20.38	0.033	0.29	1.15	28.97	1.16	5.83
7-Nov	20.50	0.031	0.30	1.15	29.01	1.16	5.68
13-Nov	21.42	0.031	0.30	1.15	29.20	1.16	5.46
17-Nov	21.57	0.030	0.31	1.15	29.45	1.17	5.33
21-Nov	21.28	0.030	0.30	1.13	29.14	1.15	5.21
25-Nov	21.64	0.030	0.31	1.15	29.53	1.17	5.10
29-Nov	21.61	0.029	0.30	1.12	29.56	1.14	4.98
3-Dec	21.52	0.028	0.28	1.04	29.53	1.05	4.91
7-Dec	21.26	0.028	0.30	1.11	29.14	1.14	4.80
11-Dec	21.04	0.026	0.28	1.06	28.83	1.08	4.66
15-Dec	21.37	0.028	0.29	1.08	29.24	1.11	4.62
19-Dec	21.16	0.027	0.29	1.09	29.07	1.09	4.55
22-Dec	20.90	0.027	0.28	1.03	28.87	1.07	4.47
27-Dec	21.02	0.028	0.28	1.03	28.83	1.07	4.39
7-Jan	21.30	0.029	0.29	1.09	29.24	1.12	4.39
14-Jan	21.32	0.03	0.34	1.10	29.10	1.17	4.20
21-Jan	25.23	0.03	0.33	1.07	33.92	1.15	4.06
28-Jan	22.19	0.03	0.33	1.08	30.08	1.16	3.91
4-Feb	21.35	0.03	0.33	1.06	29.03	1.15	3.85
11-Feb	21.45	0.03	0.33	1.06	29.27	1.14	3.74
18-Feb	21.74	0.03	0.33	1.05	29.63	1.14	3.67
25-Feb	22.15	0.03	0.34	1.05	30.09	1.14	3.50
3-Mar	21.44	0.03	0.33	1.04	29.34	1.14	3.46
10-Mar	22.76	0.04	0.33	1.05	31.07	1.15	3.44
17-Mar	21.22	0.03	0.33	1.02	29.18	1.12	3.37
24-Mar	21.85	0.04	0.32	1.03	28.81	1.11	3.27
31-Mar	21.75	0.04	0.32	1.03	28.44	1.10	3.17
7-Apr	21.23	0.04	0.33	1.04	27.92	1.13	3.11
14-Apr	21.59	0.04	0.32	1.03	28.50	1.12	2.92
21-Apr	21.28	0.03	0.31	1.00	28.02	1.08	2.85

.

Supplementary Table 2 | Saturation state of selected potential secondary minerals of the collected production well fluids from May 20, 2023 until April 21, 2024. A positive saturation state implies that the fluid is supersaturated with respect to the indicated mineral, a saturation state of zero indicates the fluid is in equilibrium with respect to the mineral phase, and a negative saturation state indicates the fluid is undersaturated. These saturation states were calculated using PHREEQC[36] together with its Kinec.v3 database[23].

Date	Ankerite	Calcite	Aragonite	Siderite	Heulandite	Montmorillonite	Chalcedony	SiO_2 (am)
20-May	-	0.21	0.06	-	0.21	2.29	0.15	-0.74
23-May	-	0.33	0.18	-	0.37	2.38	0.15	-0.74
24-May	-	0.33	0.18	-	0.29	2.36	0.15	-0.74
26-May	-	0.21	0.06	-	0.34	2.49	0.21	-0.68
29-May	-	0.24	0.09	-	-0.22	2.08	0.18	-0.71
1-Jun	-	0.09	-0.06	-	-0.37	2.03	0.20	-0.70
3-Jun	-	0.14	-0.01	-	-0.43	1.98	0.20	-0.69
5-Jun	-	0.12	-0.03	-	-0.29	2.06	0.22	-0.67
7-Jun	-	0.17	0.02	-	-0.36	2.08	0.23	-0.66
10-Jun	-	0.06	-0.09	-	-0.41	2.01	0.23	-0.66
11-Jun	-	0.17	0.02	-	-0.60	1.91	0.23	-0.66
13-Jun	-	0.01	-0.15	-	-0.63	1.90	0.26	-0.63
15-Jun	-	0.13	-0.02	-	-0.76	1.82	0.26	-0.63
17-Jun	-	-0.08	-0.23	-	-0.99	1.72	0.27	-0.62
19-Jun	-0.67	-0.04	-0.19	-1.89	-1.01	1.72	0.29	-0.60
21-Jun	-0.59	-0.01	-0.16	-1.84	-0.98	1.75	0.31	-0.58
22-Jun	-0.35	0.11	-0.04	-1.72	-0.99	1.76	0.32	-0.58
24-Jun	-0.16	0.06	-0.09	-1.48	-0.95	1.78	0.34	-0.55
27-Jun	0.00	0.05	-0.10	-1.31	-0.92	1.83	0.36	-0.53
29-Jun	0.52	0.13	-0.02	-0.87	-0.88	1.85	0.38	-0.51
1-Jul	0.63	0.07	-0.08	-0.70	-0.85	1.90	0.40	-0.49
3-Jul	0.89	0.17	0.02	-0.53	-0.87	1.87	0.40	-0.49
5-Jul	0.74	0.06	-0.09	-0.59	-0.85	1.91	0.42	-0.47
7-Jul	0.78	0.11	-0.04	-0.59	-0.89	1.84	0.42	-0.47
8-Jul	0.92	0.05	-0.10	-0.39	-0.98	1.90	0.43	-0.46
10-Jul	0.89	0.07	-0.08	-0.43	-1.00	1.85	0.44	-0.45
14-Jul	0.86	0.03	-0.12	-0.43	-0.85	1.97	0.47	-0.43
18-Jul	0.91	0.04	-0.11	-0.39	-0.84	1.97	0.48	-0.41
22-Jul	0.91	0.05	-0.10	-0.40	-0.86	1.98	0.48	-0.41
26-Jul	0.91	0.06	-0.09	-0.41	-0.82	2.02	0.49	-0.40
30-Jul	0.93	0.01	-0.14	-0.34	-0.91	1.98	0.49	-0.40
3-Aug	0.98	0.08	-0.07	-0.35	-0.72	2.09	0.50	-0.39
7-Aug	0.89	0.02	-0.13	-0.39	-0.83	2.03	0.50	-0.40
11-Aug	0.88	0.03	-0.12	-0.41	-0.76	2.07	0.50	-0.39
15-Aug	0.83	0.03	-0.12	-0.46	-0.79	2.06	0.50	-0.40
19-Aug	0.80	0.00	-0.15	-0.46	-0.80	2.06	0.50	-0.40
23-Aug	0.87	0.01	-0.14	-0.39	-0.75	2.09	0.50	-0.39
27-Aug	0.84	0.00	-0.15	-0.43	-0.75	2.08	0.50	-0.40

Supplementary Table 2 (continued) | Saturation state of selected potential secondary minerals of the collected production well fluids from May 20, 2023 until April 21, 2024.

Date	Ankerite	Calcite	Aragonite	Siderite	Heulandite	Montmorillonite	Chalcedony	SiO_2 (am)
2-Sep	0.88	0.02	-0.12	-0.39	-0.67	2.13	0.50	-0.39
4-Sep	0.88	0.02	-0.12	-0.40	-0.77	2.06	0.48	-0.41
8-Sep	0.82	-0.01	-0.16	-0.43	-0.70	2.11	0.49	-0.40
2-Oct	1.13	0.11	-0.04	-0.24	-0.73	2.11	0.46	-0.43
6-Oct	1.07	0.07	-0.08	-0.26	-0.69	2.13	0.45	-0.44
10-Oct	1.01	0.04	-0.11	-0.29	-0.63	2.17	0.47	-0.43
14-Oct	1.04	0.04	-0.11	-0.26	-0.54	2.21	0.47	-0.42
18-Oct	0.95	0.01	-0.14	-0.31	-0.64	2.15	0.47	-0.42
22-Oct	1.00	0.02	-0.03	-0.28	-0.49	2.24	0.48	-0.41
26-Oct	1.03	0.03	-0.12	-0.27	-0.42	2.29	0.48	-0.41
30-Oct	1.12	0.08	-0.07	-0.22	-0.34	2.33	0.48	-0.41
3-Nov	1.08	0.06	-0.09	-0.24	-0.38	2.30	0.48	-0.41
7-Nov	1.04	0.07	-0.08	-0.28	-0.33	2.32	0.48	-0.41
13-Nov	1.05	0.08	-0.07	-0.29	-0.26	2.36	0.48	-0.41
17-Nov	0.97	0.04	-0.11	-0.33	-0.37	2.29	0.47	-0.42
21-Nov	1.03	0.07	-0.08	-0.30	-0.24	2.37	0.48	-0.41
25-Nov	0.88	0.00	-0.15	-0.38	-0.44	2.25	0.47	-0.42
29-Nov	0.85	0.00	-0.15	-0.40	-0.69	2.09	0.43	-0.46
3-Dec	0.83	-0.02	-0.17	-0.41	-0.46	2.24	0.47	-0.42
7-Dec	0.77	-0.03	-0.18	-0.45	-0.61	2.15	0.45	-0.45
11-Dec	0.78	-0.04	-0.19	-0.44	-0.54	2.19	0.46	-0.43
15-Dec	0.79	-0.03	-0.18	-0.44	-0.56	2.18	0.45	-0.44
19-Dec	0.83	-0.01	-0.16	-0.42	-0.56	2.17	0.44	-0.45
22-Dec	0.79	-0.04	-0.19	-0.43	-0.60	2.15	0.44	-0.45
27-Dec	0.83	-0.02	-0.17	-0.41	-0.43	2.25	0.46	-0.43
7-Jan	0.80	-0.04	-0.19	-0.41	-0.30	2.33	0.48	-0.41
14-Jan	0.78	-0.02	-0.17	-0.46	-0.30	2.28	0.47	-0.42
21-Jan	0.74	-0.06	-0.21	-0.45	-0.31	2.31	0.48	-0.41
28-Jan	0.73	-0.08	-0.23	-0.45	-0.35	2.29	0.47	-0.42
4-Feb	0.50	-0.19	-0.34	-0.56	-0.57	2.17	0.47	-0.42
11-Feb	0.69	-0.10	-0.25	-0.47	-0.37	2.27	0.47	-0.42
18-Feb	0.65	-0.11	-0.26	-0.50	-0.36	2.27	0.47	-0.42
25-Feb	0.83	-0.03	-0.18	-0.40	-0.18	2.37	0.47	-0.42
3-Mar	0.96	-0.01	-0.16	-0.29	-0.13	2.39	0.47	-0.42
10-Mar	0.81	-0.04	-0.19	-0.41	-0.23	2.34	0.46	-0.43
17-Mar	1.11	0.06	-0.09	-0.20	-0.05	2.43	0.46	-0.43
24-Mar	0.89	-0.06	-0.21	-0.31	-0.28	2.31	0.45	-0.44
31-Mar	1.07	0.03	-0.12	-0.22	0.00	2.47	0.47	-0.43
7-Apr	1.26	0.13	-0.02	-0.12	0.20	2.57	0.47	-0.43
14-Apr	1.02	0.00	-0.15	-0.25	-0.03	2.45	0.46	-0.43
21-Apr	1.07	0.09	-0.04	-0.28	0.06	2.48	0.45	-0.45

Supplementary Table 3 | Saturation state of selected primary minerals of the collected production well fluids from May 20, 2023 until April 21, 2024. A positive saturation state implies that the fluid is supersaturated with respect to the indicated mineral, a saturation state of zero indicates the fluid is in equilibrium with respect to the mineral phase, and a negative saturation state indicates the fluid is undersaturated. These saturation states were calculated using PHREEQC[36] together with its Kinec.v3 database[23].

Date	Albite	Anorthite	Diopside	Enstatite	Quartz
20-May	-0.08	-4.77	-1.54	-2.24	0.40
23-May	0.00	-4.65	-1.31	-2.12	0.41
24-May	-0.02	-4.73	-1.43	-2.16	0.41
26-May	0.07	-4.94	-1.79	-2.26	0.46
29-May	-0.31	-5.38	-2.82	-2.88	0.43
1-Jun	-0.39	-5.60	-3.26	-3.08	0.45
3-Jun	-0.42	-5.69	-3.49	-3.21	0.46
5-Jun	-0.37	-5.67	-3.47	-3.20	0.48
7-Jun	-0.46	-5.77	-3.60	-3.21	0.48
10-Jun	-0.43	-5.81	-3.76	-3.33	0.48
11-Jun	-0.53	-6.01	-4.16	-3.54	0.48
13-Jun	-0.55	-6.17	-4.54	-3.73	0.51
15-Jun	-0.64	-6.33	-4.89	-3.91	0.52
17-Jun	-0.74	-6.59	-5.40	-4.16	0.52
19-Jun	-0.77	-6.73	-5.72	-4.31	0.55
21-Jun	-0.76	-6.79	-5.84	-4.37	0.56
22-Jun	-0.76	-6.81	-5.90	-4.39	0.57
24-Jun	-0.76	-6.88	-6.08	-4.48	0.59
27-Jun	-0.75	-6.98	-6.27	-4.55	0.61
29-Jun	-0.74	-7.03	-6.43	-4.63	0.63
1-Jul	-0.74	-7.10	-6.55	-4.67	0.65
3-Jul	-0.75	-7.12	-6.62	-4.72	0.65
5-Jul	-0.75	-7.20	-6.80	-4.79	0.67
7-Jul	-0.82	-7.24	-6.98	-4.93	0.67
8-Jul	-0.88	-7.38	-7.06	-4.87	0.68
10-Jul	-0.84	-7.45	-7.28	-5.02	0.69
14-Jul	-0.77	-7.43	-7.25	-4.97	0.72
18-Jul	-0.78	-7.48	-7.38	-5.04	0.73
22-Jul	-0.77	-7.51	-7.42	-5.05	0.73
26-Jul	-0.76	-7.51	-7.41	-5.03	0.74
30-Jul	-0.79	-7.60	-7.58	-5.11	0.74
3-Aug	-0.71	-7.47	-7.32	-4.97	0.75
7-Aug	-0.76	-7.56	-7.50	-5.06	0.75
11-Aug	-0.73	-7.52	-7.44	-5.03	0.76
15-Aug	-0.74	-7.52	-7.39	-4.99	0.75
19-Aug	-0.74	-7.52	-7.41	-5.00	0.75
23-Aug	-0.72	-7.49	-7.34	-4.97	0.75
27-Aug	-0.72	-7.48	-7.32	-4.96	0.75

Supplementary Table 3 (continued) | Saturation state of selected potential primary minerals of the collected production well fluids from May 20, 2023 until April 21, 2024.

Date	Albite	Anorthite	Diopside	Enstatite	Quartz
2-Sep	-0.68	-7.41	-7.19	-4.89	0.75
4-Sep	-0.73	-7.44	-7.23	-4.92	0.74
8-Sep	-0.70	-7.41	-7.17	-4.88	0.74
2-Oct	-0.70	-7.29	-6.80	-4.67	0.71
6-Oct	-0.68	-7.19	-6.58	-4.56	0.70
10-Oct	-0.73	-7.21	-6.65	-4.59	0.72
14-Oct	-0.61	-7.16	-6.57	-4.56	0.73
18-Oct	-0.66	-7.24	-6.74	-4.65	0.72
22-Oct	-0.57	-7.15	-6.56	-4.55	0.73
26-Oct	-0.54	-7.10	-6.46	-4.50	0.74
30-Oct	-0.50	-6.99	-6.24	-4.39	0.73
3-Nov	-0.52	-7.02	-6.30	-4.41	0.73
7-Nov	-0.50	-6.96	-6.20	-4.38	0.73
13-Nov	-0.47	-6.91	-6.11	-4.33	0.73
17-Nov	-0.52	-6.99	-6.26	-4.41	0.73
21-Nov	-0.45	-6.89	-6.07	-4.31	0.73
25-Nov	-0.55	-7.03	-6.34	-4.46	0.72
29-Nov	-0.65	-7.11	-6.45	-4.52	0.69
3-Dec	-0.56	-7.06	-6.39	-4.48	0.72
7-Dec	-0.64	-7.09	-6.42	-4.50	0.70
11-Dec	-0.60	-7.08	-6.42	-4.50	0.71
15-Dec	-0.60	-7.06	-6.36	-4.47	0.70
19-Dec	-0.60	-7.02	-6.28	-4.44	0.69
22-Dec	-0.62	-7.06	-6.36	-4.48	0.69
27-Dec	-0.55	-6.99	-6.25	-4.41	0.71
7-Jan	-0.49	-6.95	-6.21	0.80	0.73
14-Jan	-0.45	-6.92	-6.20	0.78	0.73
21-Jan	-0.49	-6.95	-6.21	0.74	0.73
28-Jan	-0.51	-6.97	-6.24	0.73	0.73
4-Feb	-0.62	-7.17	-6.64	0.50	0.72
11-Feb	-0.52	-6.97	-6.24	0.69	0.72
18-Feb	-0.51	-6.96	-6.24	0.65	0.72
25-Feb	-0.42	-6.77	-5.85	0.83	0.72
3-Mar	-0.39	-6.75	-5.83	0.96	0.73
10-Mar	-0.45	-6.79	-5.88	0.81	0.71
17-Mar	-0.36	-6.59	-5.47	1.11	0.71
24-Mar	-0.48	-6.80	-5.88	0.89	0.71
31-Mar	-0.35	-6.58	-5.46	1.07	0.72
7-Apr	-0.24	-6.38	-5.05	1.26	0.72
14-Apr	-0.36	-6.59	-5.47	1.02	0.71
21-Apr	-0.31	-6.42	-5.11	1.07	0.70

Supplementary Data Table 4 | Summary of powdered diffraction files (pdf) used in the identification of minerals in Supplementary Fig 1 and Extended data Table 3.

Mineral	PDF File	Mineral	PDF File
Albite (ordered)	09-0466	Gypsum	33-0311
Ankerite	84-2066	Lepidocrocite	44-1415
Augite	70-3753	Magnetite	19-0629
Biotite	76-0884	Microcline	19-0926
Calcite	05-0586	Quartz	46-1045
Clinochlore	24-0506	Richterite	81-0724
Goethite	81-0463	Siderite	29-0696
